# Calibration-free PAT: Locating selective crystallization or precipitation sweet spot in screenings with multi-way PARAFAC models

**DOI:** 10.3389/fbioe.2022.1051129

**Published:** 2022-12-14

**Authors:** Christina Henriette Wegner, Jürgen Hubbuch

**Affiliations:** Institute of Process Engineering in Life Sciences, Section IV: Biomolecular Separation Engineering, Karlsruhe Institute of Technology (KIT), Karlsruhe, Germany

**Keywords:** selective precipitation, selective crystallization, multi-way chemometrics, parallel factor analysis (PARAFAC), ultravioletvisible light (UV/Vis) spectroscopy, high-throughput (HT) screening, calibration-free

## Abstract

When developping selective crystallization or precipitation processes, biopharmaceutical modalities require empirical screenings and analytics tailored to the specific needs of the target molecule. The multi-way chemometric approach called parallel factor analysis (PARAFAC) coupled with ultraviolet visible light (UV/Vis) spectroscopy is able to predict specific concentrations and spectra from highly structured data sets without the need for calibration samples and reference analytics. These calculated models can provide exploratory information on pure species spectra and concentrations in all analyzed samples by representing one model component with one species. In this work, protein mixtures, monoclonal antibodies, and virus-like particles in chemically defined and complex solutions were investigated in three high-throughput crystallization or precipitation screenings with the aim to construct one PARAFAC model per case. Spectroscopic data sets of samples after the selective crystallization or precipitation, washing, and redissolution were recorded and arranged into a four-dimensional data set per case study. Different reference analytics and pure species spectra served as validation. Appropriate spectral preprocessing parameters were found for all case studies allowing even the application of this approach to the third case study in which quantitative concentration analytics are missing. Regardless of the modality or the number of species present in complex solutions, all models were able to estimate the specific concentration and find the optimal process condition regarding yield and product purity. It was shown that in complex solutions, species demonstrating similar phase behavior can be clustered as one component and described in the model. PARAFAC as a calibration-free approach coupled with UV/Vis spectroscopy provides a fast overview of species present in complex solution and of their concentration during selective crystallization or precipitation, washing, and redissolution.

## 1 Introduction

The variety and number of biopharmaceutical products are constantly increasing. There are e.g., monoclonal antibodies (mAbs) (Elvin et al., 2013), vaccines (Lua et al., 2014; Saxena et al., 2021), and new therapeutics (Tobin et al., 2014). Each new therapeutic drug is accompanied by new physico-chemical properties, which need to be assessed with target molecule-specific analytics to ensure drug purity and safety for the patient. Broadly applicable analytical technologies are preferred as they can characterize various products and process steps. This may lead to deeper product and process knowledge, together with cost- and risk-based decisions during process development.

Downstream processes of biopharmaceutical products commonly rely on preparative chromatographic processes, which are costly or difficult to scale-up. In general, selective protein crystallization or precipitation can be an alternative to costly chromatography capture steps (Perosa et al., 1990; McDonald et al., 2009; Smejkal et al., 2013) and bear their advantages, e.g., high purity, concentration, and stability during product storage (dos Santos et al., 2017; Roque et al., 2020). Given that the process conditions are selected appropriately, these processes can provide highly concentrated products and can be scaled at lower costs compared to chromatographic process steps.

To speed up the process of finding optimal process conditions, empirical high-throughput (HT) studies are common for early-stage process development and require HT-compatible analytics. In this context, fast, non-destructive, versatile methods, e.g., spectroscopic methods, are preferred and they can be used to determine critical process parameters, e.g., target protein concentration, yield, and purity.

When combining HT studies and spectroscopy, though, a situation often arises where large data sets are recorded which are difficult to interpret and are strongly correlated; the information sought-after is hidden in a data jungle. To overcome these limitations, scientists commonly apply chemometric methods to large spectral data sets, e.g., partial least squares (PLS) regression (Saleemi et al., 2012; Simone et al., 2014; Rüdt et al., 2017), convolutional neural networks (CNNs) (Acquarelli et al., 2017), or Gaussian process regression (Chen et al., 2007), and generate process analytical technology (PAT) models to improve the design, analysis, and control during product manufacturing (Rathore et al., 2010). The mentioned regression models, however, generally require robust reference analytics for calibration. Specific PAT research on crystallization processes mainly focused on mechanistic models for crystal nucleation or growth implementing physical or empirical equations and is discussed elsewhere (Szilagyi et al., 2020; Trampuž et al., 2020, 2021).

In the case of spectroscopy measurements recorded over time, three-dimensional (3D) data sets are generated, which are ordered along three dimensions, e.g., wavelength, time, and absorbance. When the spectra of several samples are recorded, four-dimensional (4D) data sets are formed. This multi-dimensionality further complicates the data analysis and calls for multi-way chemometrics. To process data sets of higher order, multi-way chemometric approaches, e.g., generalized rank annihilation method (GRAM), unfolded partial least-squares (U-PLS), and multi-way partial least-squares (N-PLS) regression models, require external calibration (Olivieri, 2014; Anzardi et al., 2021). They cannot be applied when accurate reference analytics are missing, e.g., in product capture process steps due to the variety of product- and process-related impurities.

On the contrary, parallel factor analysis (PARAFAC) models can analyze data sets of higher order without the need for calibration samples. Given the number of components in the data set, the PARAFAC model can decompose a linear, spectral data set of second or higher order into the signal contribution of each component and regress the model towards a minimal model error compared to the original data set. In this application, one PARAFAC component represents one species in the data set. As a result, the initial data set can be described as the sum of loading vectors of each species in each dimension and the model error (Bro, 1997; Levi et al., 2004; Yu et al., 2021). PARAFAC was successfully applied to qualitative and quantitative data analysis on excitation emission spectra of fluorescence spectroscopy (Andersen and Bro, 2003; Ortiz et al., 2015; Steiner-Browne et al., 2019) using data sets structured along excitation wavelength x emission wavelength x samples. Other possible applications are the flow injection analysis (FIA) (Marsili et al., 2004; Niazi et al., 2005) and high-performance liquid chromatography (HPLC) runs equipped with multi-variate detector, e.g., diode array detector (DAD) (Leitão and Esteves Da Silva, 2006; García et al., 2007) or mass spectrometry (MS) (Ortiz et al., 2015; Ortiz et al., 2020).

The mentioned work on PARAFAC models focused on the deconvolution of overlapping peaks in chromatography runs or the quantification of chemical analytes in fluorescence spectroscopy. With regard to the rising number of new biopharmaceuticals and early stage process development, HT screenings for crystallization and precipitation processes are time-consuming and need to be evaluated quickly with versatile analytics.

This calls for the investigation of the PARAFAC model application to identify sweet spots in the phase behavior of biopharmaceuticals for crystallization or precipitation processes. This research project thus investigates how PARAFAC models can predict specific spectra and concentration profiles in a screening of unknown species from ultraviolet visible light (UV/Vis) data. To show the broad applicability of PARAFAC to HT screenings, three case studies on phase behavior were conducted. The case studies covered one selective protein crystallization process of a defined ternary protein system and two selective precipitation processes of mAbs and virus-like particles (VLPs) in complex solutions. Depending on the case study, UV/Vis spectra were recorded from supernatant samples taken from different process steps, e.g., crystallization, precipitation, washing, and redissolution. Time-resolved spectroscopic data were obtained by injecting samples into a HPLC system equipped with a DAD. No chromatographic column was installed to save analysis time and generate the data with a universal method unaffected by the investigated molecule. This analytical setup led to a second-order data set of three dimensions (wavelength x time x samples). The PARAFAC model calculated the loadings in the mentioned dimensions for each component describing the spectral, time, and concentration profile of the different species.

The presented results demonstrate how multi-way chemometrics can explore spectroscopic screening data sets of higher order. Different case studies with varying product characteristics may be examined with little experimental effort and in a calibration-free way. The PARAFAC models can help to assess selective crystallization and precipitation conditions with regard to purity and yield while increasing process knowledge in early stage process development of new biopharmaceutical products. Reference analytics for calibration are not required for the model calculation making it suitable for use in early stage process development. Additionally, qualitative information on spectra and phase behavior increase process knowledge and may be used for process development according to quality by design (QbD).

## 2 Material and methods

**FIGURE 1:**
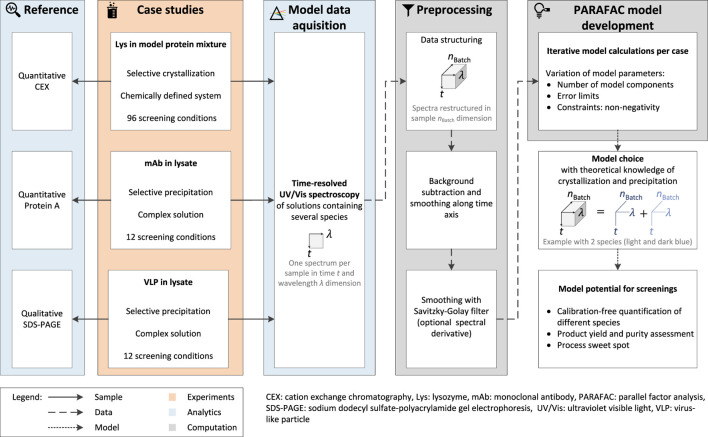
The workflow for the PARAFAC model calculation can be divided into the experimental work of three different case studies, the analytics, and the computational work. Screening samples are UV/Vis-analyzed and the recorded spectral data set is restructured in the dimensions time *t*, wavelength *λ*, and supernatant sample *n*_Batch_. Subsequent preprocessing allowed the calculation of one PARAFAC model per case study. The reference analytics validate the generated models and vary depending on the target molecule, purification process, i.e., selective crystallization or precipitation, and the composition of the initial material.

The preparation and execution of the first case study were described in detail by Wegner et al. (2022) and are described in brief in this work. An overview of the experimental setup, analytics, and computation is visualized in Figure 1.

### 2.1 Experiment buffer and protein preparation

All chemicals were purchased from Merck KGaA (Darmstadt, DE), unless otherwise stated. The buffer solutions were prepared at room temperature with ultrapure water (PURELAB Ultra, ELGA LabWater, Lane End, High Wycombe, U.K.), pH-adjusted with 32% hydrochloric acid (HCl) or 4 mol sodium hydroxide (NaOH).

In the first case study, lyophilized model proteins lysozyme (Lys) from chicken-egg-white (Hampton Research, Aliso Viejo, CA), ribonuclease A (RibA) from bovine pancreas, and cytochrome C (CytC) from equine heart were dissolved in multi-component buffer (MCB, 21 mmol N-1,1- dimethyl-2-hydroxyethyl-3-amino2-hydroxypropanesulfonicacid (AMPSO), 17 mmol 3-N-morpholino propansulfonic acid (MOPS, Carl Roth GmbH + Co. KG, Karlsruhe, DE), 15 mmol succinate acid AppliChem GmbH, Darmstadt, DE) at pH 9. After dialysis to the target multi-component buffer (MCB), the protein concentrations were adjusted as required and the protein solutions were filtered (0.2 µm, Pall Corporation, Port Washington, NY).

For the second case study, Byondis B.V. (Nijmegen, NL) kindly provided frozen cell culture supernatant (CCS) of a mAb harvest of chinese hamster ovary (CHO) cells. The material was thawed, filtered (0.2 µm, Pall Corporation), aliquoted, and stored at −20◦ until later usage. The required amount of CCS was thawed and a buffer exchange was performed to a phosphate-buffered saline (PBS) buffer [58.4 mmol sodium chloride (NaCl), 74.6 mmol potassium chloride (KCl), 136.1 mmol potassium dihydrogenphosphate (KH2PO4), 142.0 mmol disodium hydrogen phosphate (Na2HPO4), pH 7.4] using a PD MiniTrap^TM^G-25 column (GE Healthcare, Chicago, IL). The CCS stock solution was filtered (0.2 µm, Pall Corporation) prior to screening.

The third case study involved truncated Hepatitis B core antigen (HBcAg) VLPs (Zlotnick et al., 1996). The VLPs were produced in-house in *E. coli* as previously described by Hillebrandt et al. (2020). After filtering the lysed material with a glass fiber, a 0.45 µm, and a 0.2 µm cellulose acetate (CA) syringe filter (Sartorius Stedim Biotech GmbH, Göttingen, DE), the material was 3x diluted, aliquoted, and stored at −30◦ until further usage. For the screenings, the material was thawed and filtered (0.2 µm, CA, Pall Corporation).

The used crystallization solution was the MCB at pH 9 and contained additional 3.5 mol ammonium sulfate (AMS). The precipitation solution of the second and third case studies contained only 3.6 mol AMS. The redissolution buffers were PBS buffer, pH 7.4 in the second (mAb) and 50 mmol Tris buffer, pH 7.2 in the third case study (VLP).

### 2.2 Crystallization and precipitation experiments

The following subchapter describes the experimental conditions of the three HT screening case studies. The second and third paragraphs deal with selective crystallization in a ternary protein mixture and with the selective precipitation of mAbs and VLPs in complex solutions, respectively.

The prepared protein solutions for the ternary phase diagram were mixed and crystallized in 24 µl micro-batches as described by Wegner et al. (2022). 3 µl samples for the analysis were drawn after 13 days of incubation at 8°C and 50 times diluted with MCB, pH 9.

The selective precipitation screenings were conducted by mixing 278 µl of 12 differently diluted precipitation solutions with 222 µl of the initial mAb or VLP protein stock solutions leading to twelve 500 µl batches. The desired screening range of AMS was between 0 and 2 mol. The precipitation solutions were shaken using a thermo shaker at 300 rpm for 30–60 min and then centrifuged (17000 g, 2 min). The shaking and centrifugation conditions were used for all steps. The supernatant (S1) was removed, and a wash step was performed by adding 500 µl of a buffer containing the same components as the respective screening condition. Then, the supernatant solutions were centrifuged and the wash step supernatant (S2) was removed. Adding 500 µl of the respective redissolution buffer (see Subchapter 2.1) and shaking for 2 h redissolved the precipitate. Eventually, the redissolution batches were centrifuged (S3).Supernatant samples (S1–S3) were drawn after each centrifugation step, diluted (mAb: 2 times; VLP: 10 times) with redissolution buffer, and cooled at 8°C until the analysis at the end of the experiment.

### 2.3 Analytics

#### 2.3.1 Multi-way UV/Vis spectra

First, the samples were UV/Vis-analyzed using a Dionex Ultimate 3000 RS HPLC system (Thermo Fisher Scientific, Inc., Waltham, MA) equipped with a RS diode array detector. The UV/Vis spectra were recorded by injecting 20 µl sample volume into the device with no column installed. The injection volume stayed constant for all HPLC measurements. The detector data acquisition was performed with 100 Hz frequency and in the wavelength range of 240 nm–450 nm for the first and 220 nm–550 nm for the remaining case studies. A filter cartridge (pore size 0.5 µm, OPTI/SOLV EXP, Merck KGaA (Darmstadt, DE)) was integrated to impede aggregates in the detector. The mobile phase was a (50 mmol Tris, 100 mmol NaCl, pH 8.0) buffer for the first case study or the respective redissolution buffer of the case study and the flow rate was 200 μl min^−1^ in the first or 50 μl min^−1^ for the remaining case studies.

#### 2.3.2 Reference analytics

Different analytics were applied depending on the case study and target protein. The reference data of the first study were derived from cation exchange chromatography (CEX) performed with a ProSwift SCX-1S 4.6 × 50 mm column using the aforementioned HPLC system (see Subchapter 2.3.1 with a low salt buffer (50 mmol Tris, pH 8.0) and high salt buffer (50 mmol Tris, 1 mol NaCl, pH 8.0) with a flow rate of 1.5 ml min^−1^ (Wegner et al., 2022). A 2.1 × 30 mm POROS™ protein A column (Applied Biosystems, Waltham, MA) was used to separate the mAbs from the contaminants, and it allowed species quantification. After sample injection, the column was equilibrated with equilibration buffer (PBS buffer, pH 7.4) for 16  column volumes (CVs) and eluted with elution buffer (PBS buffer, pH 2.6) for 28 CVs. The flow rate was set to 2 ml min^−1^. For the third case study, the sample purity was assessed only qualitatively with sodium dodecyl sulfate–polyacrylamide gel electrophoresis (SDS-PAGE). The analysis was performed with lithium dodecyl sulfate (LDS) sample buffer, 2-(N-morpholino)ethanesulfonic acid (MES) running buffer, and NuPage 4–12% BisTris Protein Gels (all Thermo Fisher Scientific, Inc.). The addition of reducing 50 mmol dithiothreitol (DTT) was the only adaption to the manufacturer’s protocol.

The pure species spectra of Lys, RibA, and CytC were recorded by measuring single protein solutions using the setup described in Subchapter 2.3.1. In line with this, the pure VLP spectrum was derived from a re-dissolved and sterile-filtered VLP solution purified by diafiltration and multimodal size-exclusion chromatography according to Hillebrandt et al. (2021). The contaminant and the pure mAb species spectra were calculated from the protein A analysis flow-through and elution peak.

### 2.4 Data analyses

All data analyses, preprocessing, and model calibration were performed in MATLAB, R2019b (The MathWorks, Inc., Natick, MA), including the MATLAB N-way toolbox (Andersson and Bro, 2000) to construct the chemometric models.

#### 2.4.1 Data structure and preprocessing

Each UV/Vis-analyzed sample measurement led to a 3D spectral data set spanned over the system retention time, wavelength measuring the absorbance, similar to a 3D chromatographic data set with strongly overlaying species peaks. When multiple supernatant samples per case study were analyzed, the generated data were arranged along the sample number leading to a 4D data set. For each case study, one 4D data set was constructed, preprocessed, and used for the model calculation. Preprocessing (see Figure 1) consisted of the background subtraction and smoothing the absorbance data set along the time axis. The preprocessed data were cut to a wavelength range of 255 nm–410 nm for the first and 255 nm–310 nm for the remaining case studies to leave out the non-absorbing wavelength ranges and thus improve the model development. For each case study, the preprocessing parameters were varied and tested for the spectral and time-wise smoothing (see Table 1) with a Savityky-Golay smoothing filter (Savitzky and Golay, 1964). The third data set required the calculation of the second derivative with the Savitzky-Golay filter to enhance spectral differences as the species present in the examined solutions showed strongly overlapping spectra.

**TABLE 1:** Preprocessing and model development parameters: These parameters were varied for each case study to find optimal calculation parameters. The final calculation parameters are listed as well.

		Data preprocessing			Model parameters	
		Derivative	Time smoothing range	Wavelength smoothing range	Number of model components	Error limit
Case 1	max	2	10	13	3	0.010000
	min	0	1	3	2	0.000001
Case 2	max	0	51	7	4	0.008000
	min	0	10	5	3	0.000010
Case 3	max	2	35	7	4	0.008000
	min	0	10	5	2	0.000100
Case 1	final	0	3	7	2	0.000001
Case 2	final	0	10	7	3	0.000100
Case 3	final	2	10	7	3	0.000100

#### 2.4.2 PARAFAC model construction

The calculation of the PARAFAC models (see Figure 1) was performed varying the model parameters, i.e., error limits, and number of PARAFAC components. Especially, the latter needs to be selected with care as this parameter is essential for a valid model. These model calculation parameter ranges are listed in Table 1. Additionally, the non-negativity constraint was imposed in time, wavelength, and concentration dimension in all case studies with one exception. For the third model, this constraint was left out in the wavelength dimension due to the second-derivative preprocessing data treatment (see Subchapter 2.4.1). Due to instability reasons of the PARAFAC model algorithm, ten different models for each selected preprocessing and model parameter set were calculated. The model with the highest core consistency diagnostic (CORCONDIA) value (Bro and Kiers, 2003) was chosen if the loadings in the concentration mode were sensible and agreed with the theoretical knowledge of protein crystallization and precipitation. In detail, this means that the calculated concentration loadings of all protein species were assumed to decrease to their protein-specific solubility lines with increasing precipitant concentration. The inverse behavior was expected for the analyzed redissolution solutions.

The used PARAFAC algorithm kept the data variance only in the first mode - the time loadings–leading to normalized spectral and concentration loadings.

## 3 Results

### 3.1 Case 1–selective crystallization of lysozyme in a ternary protein solution

As a proof of concept, the PARAFAC model construction was first applied to UV/Vis spectral data of a phase transition process of a chemically defined system. In a system of three model proteins, the target molecule (Lys) was selectively crystallized in a HT screening with 96 different conditions. The other two species (CytC and RibA) are arbitrarily treated as contaminants and were preferred to stay in the supernatant to achieve a high Lys purity in the crystals. The supernatants of the screened conditions holding different protein-specific concentrations were UV/Vis-analyzed. The generated data was used for the model construction. The selected model required two PARAFAC components–one for the target molecule Lys, and the second one for clustering the contaminants.

**FIGURE 2:**
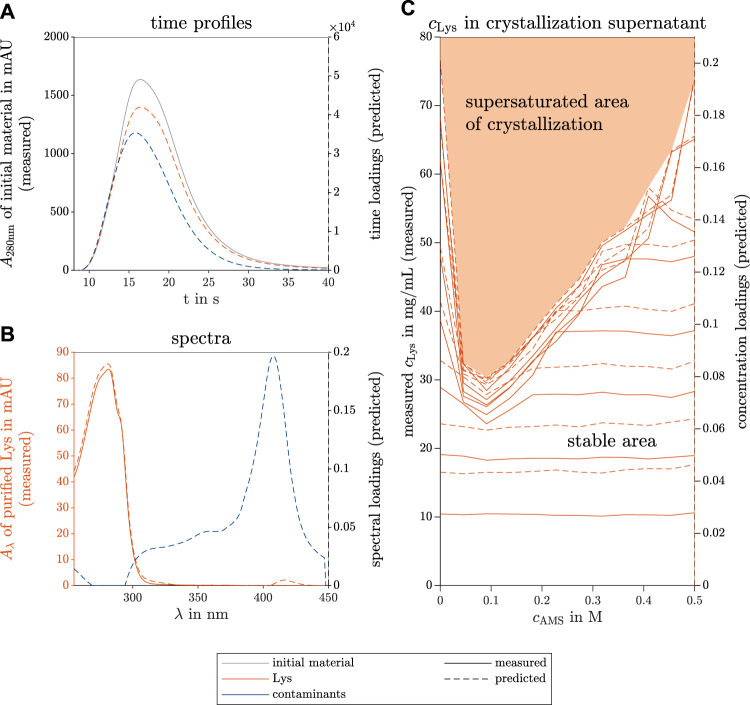
PARAFAC model results of the selective crystallization screening of Lys in a ternary model protein system. The measured reference data (left axis) and the predicted loadings (right axis) are illustrated with solid and dashed lines, respectively. The colors gray, orange, and blue indicate the initial raw material, the target species Lys, and the contaminating species, respectively. The time course loadings in **(A)** show the PARAFAC model predictions of the species absorption loadings over time *t* in the flow cell of the UV/Vis detector. Additionally, the spectral absorption of the initial solution *A*_280nm_ is shown at wavelength 280 nm over time. The spectral loadings in **(B)** demonstrate the similarity between the predicted and the measured Lys absorption spectra *A*_*λ*_ over the wavelength *λ*. From the concentration loadings in **(C)**, the predicted saturation curve can describe the phase behavior of Lys in the investigated ternary model system and can distinguish the screened conditions into the supersaturation and stable area. The variables *c*_Lys_ and *c*_AMS_ represent the concentrations of Lys and AMS, respectively.

Figure 2A shows the PARAFAC-predicted single species time profiles compared to the measured absorbance of the initial material at the wavelength *λ* = 280 nm over time. The dashed and solid lines visualize the model-predicted data (right axis) and the measured data (left axis), respectively. This remains consistent throughout this research work. The predicted spectra of the two components are illustrated in Figure 2B in different colors for each species. As a reference, the pure Lys spectrum is included with solid lines for identification of the target molecule component. The predicted and measured Lys concentration of the supernatants of the screened conditions are depicted in Figure 2C. This plot illustrates the phase behavior of Lys in a phase diagram depending on the AMS and initial Lys concentration of the screened condition, and distinguishes between the supersaturation and stable area. The loading vectors in all three modes are unitless, and one component represents one species in each mode. The concentration of the contaminant species did not change (data not shown). The phase behavior of this HT screening is described and explained in detail by Wegner et al. (2022).

The time courses of the predicted two species match the position of the overall absorbance at *λ* = 280 nm of the analyzed initial material. Both predicted species demonstrate a similar flow behavior through the HPLC system during the no-column runs and resemble the Gaussian shape due to axial diffusion in the analysis system. The spectral prediction of the Lys component fits the measured spectrum of pure Lys, only the shoulder at *λ* = 290 nm is slightly less pronounced than in the measured spectrum. The predicted concentration loadings and measured concentrations overlay and indicate the saturation curve of the phase diagram clearly. This curve distinguishes the screened condition into the stable area showing no Lys concentration decline in the supernatant and the supersaturation area, in which the Lys concentration drops to the saturation curve, possibly due to crystallization.

**FIGURE 3:**
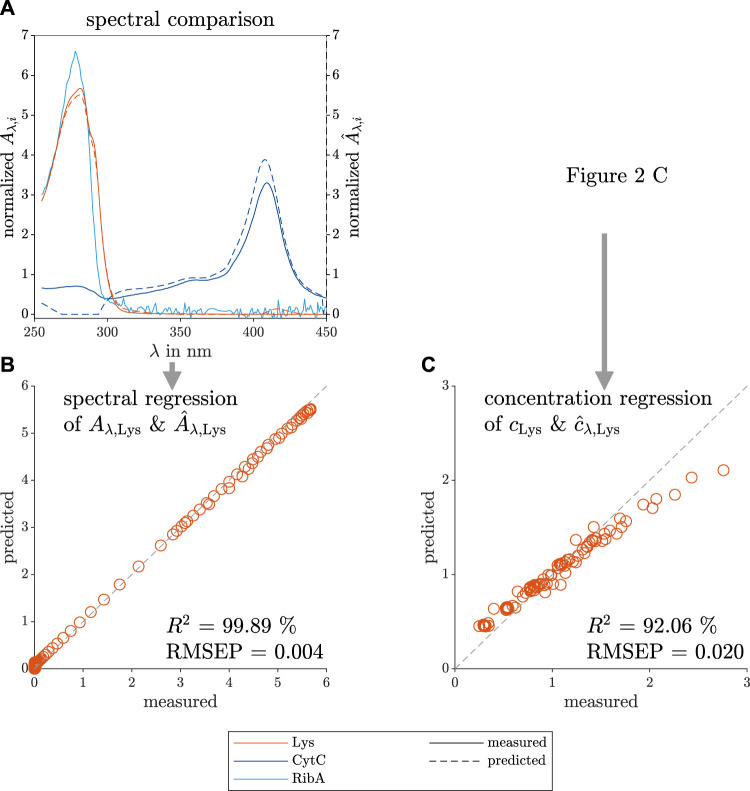
Comparison between predicted and measured data of the spectral and concentration loadings of the selective crystallization screening of Lys. The measured reference data (left axis) and the predicted loadings (right axis) are illustrated with solid and dashed lines, respectively. The colors orange, dark blue, and light blue indicate the contaminating species, target species Lys, the model proteins CytC and RibA, respectively. The predicted spectral loadings, and the measured reference data are used to calculate the mean-normalized predicted and measured absorption ( ${\hat{A}}_{\lambda ,i}$ & *A*_*λ*,*i*_) which are plotted over the wavelength *λ* for each species *i* in **(A)**. The predicted absorption ${\hat{A}}_{\lambda ,Lys}$ of Lys is shown over the measured absorption *A*_*λ*,Lys_ of Lys in **(B)**. Figure 2C is used to calculate the mean-normalized predicted concentration loadings ${\hat{c}}_{\text{Lys}}$ and the measured concentration data *c*_Lys_ of Lys in **(C)**. The gray dashed lines visualize the ideal fit of the predicted to the measured data **(B,C)**. The calculated, high coefficient of determination *R* ^2^ values support the PARAFAC model validity.

To compare the predicted PARAFAC loadings and the measured reference data, Figure 3 depicts the model and measurement data sets in two ways. First, the data sets in Figure 3A show the predicted spectral loadings and measured species, similarly to Figure 2B, but with the spectra of all three model proteins (Lys, CytC, and RibA) present in the screening solutions. Second, the spectral data of the Lys species were mean-normalized to overcome the difference in axis scale. Finally, the data sets were plotted against each other and used for the coefficient of determination (*R* ^2^) and root mean squared error of prediction (*RMSEP*) calculation (see Figure 3B for the Lys spectrum and Figure 3C for the concentration comparison). Figure 3C is derived from the mean-normalized concentration data of Figure 2C. The *RMSEP* in this work is given without a unit as the variable is calculated from normalized values.

The RibA UV/Vis spectrum shows a noisy spectrum above 300 nm, which is a normalization artefact as the overall absorption of the pure RibA spectrum was low due to its low extinction coefficient and the measured concentration of 0.2 mg ml^−1^. It is visible that the predicted contaminant spectrum is similar to the pure CytC spectrum between 300–450 nm. According to the model, below 300 nm, the two contaminant species (CytC and RibA) do not contribute to the measured UV/Vis absorbance which differs from the measured pure species spectra. PARAFAC models with three components did not lead to reasonable models, so that the species RibA was not modeled as an own species due to its low contribution to the overall UV/Vis absorbance. However, RibA and CytC together can be clustered as impurities and can be described by one contaminant component as they demonstrate similar phase behavior.

The mean-normalized model prediction and the measured mean-normalized spectrum of pure Lys overlay as indicated by the high *R* ^2^ value. The Lys concentration loadings of the PARAFAC model are slightly underestimated at higher protein concentrations, which is quantified with a lower *R* ^2^.

### 3.2 Case 2–selective precipitation of monoclonal antibodies in a complex solution

As the second case study, a mAb was selectively precipitated out of a clarified, complex solution (CCS) consisting of several different species. In total, 12 different precipitant concentrations were investigated, and the supernatants of the precipitation (S1), wash (S2), and redissolution (S3) process steps were UV/Vis-analyzed to finally construct a valid PARAFAC model.

**FIGURE 4:**
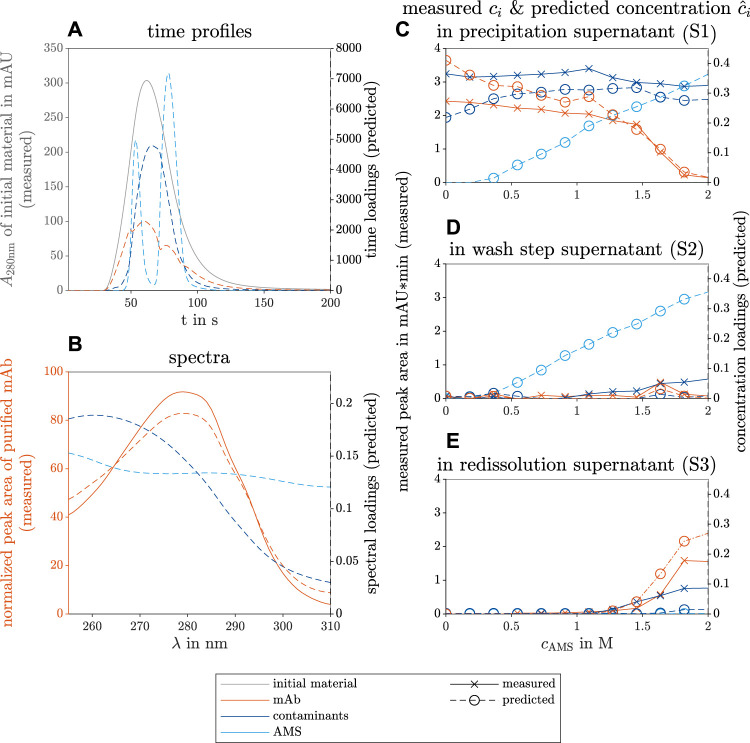
PARAFAC model results of the selective mAb precipitation screening from clarified CHO CCS. The measured reference data (left axis) and the predicted loadings (right axis) are illustrated with solid and dashed lines, respectively. The colors gray, orange, dark blue, and light blue indicate the initial raw material, the target mAb, the contaminating species, and the precipitant AMS. The time course loadings in **(A)** show the PARAFAC model predictions of the species absorption loadings over time *t* in the flow cell of the UV/Vis detector. Additionally, the spectral absorption of the initial solution *A*_280nm_ is shown at wavelength 280 nm over time. The spectral loadings in **(B)** illustrate the predicted contaminant spectrum over the wavelength *λ* and the similarity between the predicted and the measured mAb spectrum. The predicted concentration loadings ${\hat{c}}_{i}$ are shown over varying precipitant concentration *c*_AMS_ during the precipitation in **(C)**, wash step **(D)**, and redissolution process step **(E)**. The measured concentration *c*_*i*_ is derived from the peak area of the reference analytics. The peak areas of a reference analytic represent the concentrations of the mAb and the contaminant. They are shown in **(C–E)** with solid lines.

The results of the constructed model with three different components are shown in Figure 4. The three components could be identified as the mAb, contaminants, and AMS. The predicted time profiles of each component and the measured absorbance at *λ* = 280 nm are shown in Figure 4A. The predicted spectral profiles and the measured spectrum of purified mAb are depicted in Figure 4B. The predicted, specific concentration in the supernatant of precipitation (Figure 4C), wash (D), and redissolution process step (E) are colored according to the species. As a reference, the measured peak area of the mAbs and the contaminant are included in Figures 4C–E and represent the concentration profile throughout the investigated screening conditions.

The predicted time profiles in Figure 4A show a Gaussian curve for the contaminant species, two Gaussian curves for the AMS species, and an irregular profile for the mAb component resembling multiple overlaying species. The predicted AMS time profile overlaps with the measured time profiles of pure AMS solution measurements (see Supplementary Figure S1).

The predicted spectrum of the target molecule mAb fits the measured spectrum of protein A purified mAb (see Figure 4B). The predicted concentration profile of the AMS during the precipitation and wash step agrees with the experimental AMS concentration as the precipitant concentration was linearly increased over the investigated conditions from 0 mol to 2 mol during the precipitation and wash process step (see Supplementary Figure S2). The predicted and the measured mAb concentrations in the precipitation supernatants decrease strongly above 1.2 mol AMS in Figure 4C and match the increase in mAb concentration in the redissolution solutions above the same AMS concentration in Figure 4E. The predicted and the measured contaminant concentrations behave likewise with a different threshold at 1.6 mol AMS. A slight increase in the mAb concentration at 1.6 mol AMS during the washing step is visible in the predicted and the measured data sets. A slight increase in the contaminant concentration with rising AMS concentration was only seen in the reference analytics and indicates contaminant removal during the wash step. The predicted mAb concentration in Figure 4C is overestimated at AMS concentration between 0 and 0.4 mol AMS whereas the contaminant concentration is underestimated. Similarly, the behavior of overestimated mAb and underestimated contaminant concentrations is visible in the redissolution samples at higher AMS screening conditions in Figure 4E.

**FIGURE 5:**
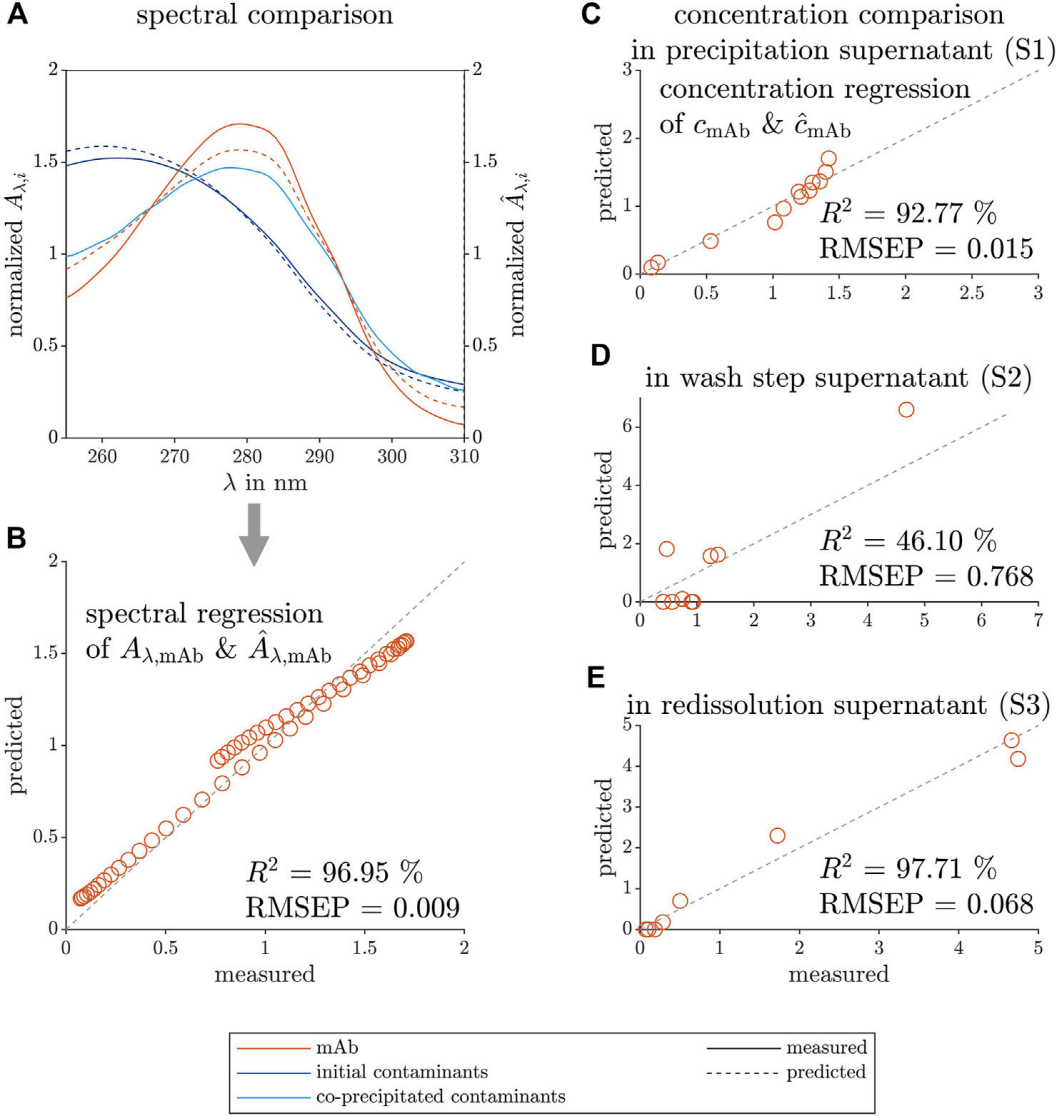
Comparison between predicted and measured data of the spectral and concentration loadings of the selective mAb precipitation screening. The measured reference data (left axis) and the predicted loadings (right axis) are illustrated with solid and dashed lines, respectively. The colors orange, dark blue, and light blue represent the target species mAb, the contaminating species before precipitation, and the remaining contaminant species after redissolution, respectively. The spectral predictions ${\hat{A}}_{\lambda ,i}$ and measurements *A*_*λ*,*i*_ are mean-normalized and depicted over the wavelength *λ* in **(A)**. The predicted spectral mAb loadings ${\hat{A}}_{\lambda ,mAb}$ and the measured reference spectrum of purified mAb *A*_*λ*,mAb_ are used to plot the predicted over measured data in **(B)**. The predicted mAb concentration loadings ${\hat{c}}_{\text{mAb}}$ and measured concentration reference *c*_mAb_ from mAb peak areas see **(C–E)** are mean-normalized and plotted against each other for the process steps of precipitation **(C)**, washing **(D)**, and redissolution **(E)**. These data were used to calculate the coefficient of determination *R* ^2^ values to quantify the validity of the constructed model. The gray dashed lines visualize the ideal fit of the predicted to the measured data **(B–E)**.

To further validate the constructed PARAFAC model, comparisons of the predicted loadings, and measured data of the mAb spectrum and concentration are illustrated in Figure 5. The predicted spectral loadings of the mAb and the predicted contaminant are shown in Figure 5A, as well as the spectrum of the initial contaminants, present in the precipitation supernatant, and of the co-precipitated contaminants, which are still present after redissolution. The initial contaminants, which are present in large excess and remain in solution despite the presence of the precipitant AMS, are well described by the blue contaminant component of the PARAFAC model. The co-precipitated contaminants could not be described by the model as these contaminants underwent phase transition at similar precipitant concentration as the target molecule. The mean-normalized, predicted spectral loadings and the measured spectrum of the mAb species are depicted in Figure 5B and agreed as indicated by the *R* ^2^ value of 97.38% and a low *RMSEP* of 0.009.

To further visualize the model agreement, the predicted, mean-normalized concentration loadings and measured peak area of the mAb are shown during the different process steps in Figures 5C–E with their process-specific *R* ^2^ and *RMSEP* values. The concentration loadings show moderate agreement with the measured data for the precipitation and wash step samples. In the precipitation supernatant analysis, the presence of the different contaminants at high mAb concentration (especially at lower AMS concentration) might be the cause. The wash step analysis samples showed very low mAb concentration except for one outlier. The lowest *R* ^2^ and the highest *RMSEP* values among the investigated process steps might be caused by a mathematical artefact and the outlier. The high *R* ^2^ and low *RMSEP* values for the precipitation and redissolution supernatant indicate that the model could produce valid mAb concentrations.

### 3.3 Case 3–selective precipitation of virus-like particles in a complex solution

The third case study dealt with the selective precipitation of VLPs in *E.coli* lysate. In line with the second case study, a screening was performed over different precipitant concentrations, and the UV/Vis-analyzed precipitation (S1), wash (S2), and redissolution step (S3) supernatants were used to construct a PARAFAC model.

**FIGURE 6:**
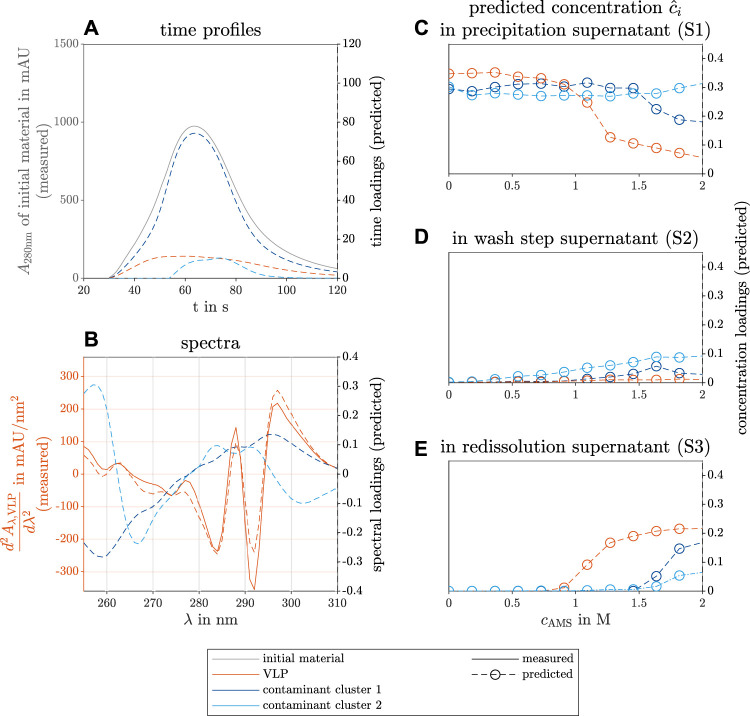
PARAFAC model results of the selective VLP precipitation screening from *E.coli* lysate. The measured reference data (left axis) and the predicted loadings (right axis) are illustrated with solid and dashed lines, respectively. The colors gray, orange, dark blue, and light blue indicate the initial raw material, the VLPs, and two contaminant clusters. The time course loadings in **(A)** show the PARAFAC model predictions of the species absorption loadings over time *t* in the flow cell of the UV/Vis detector. Additionally, the spectral absorption of the initial solution *A*_280nm_ is shown at wavelength 280 nm over time. The spectral loadings in **(B)** illustrate the predicted contaminant spectra over the wavelength *λ* and the similarity between the predicted loadings and the measured second derivative of the VLP spectrum $\frac{d^{2}A_{\lambda ,VLP}}{d\lambda^{2}}$ . The predicted concentration loadings ${\hat{c}}_{i}$ are shown over varying precipitant concentration *c*_AMS_ during the precipitation in **(C)**, wash step **(D)**, and redissolution process step **(E)**.

The results of the constructed model with three different components are shown in Figure 6. The three components are identified as the VLPs and two contaminant clusters.

The time profiles in Figure 6A show a flat, broad peak for the VLP species. The calculation of the second derivative of the spectra along the wavelength dimension improved the model validity (data not shown). The second spectral derivative of a reference spectrum of purified VLPs validated the spectral PARAFAC loadings (see Figure 6B). The reference data illustrate how well the peak position is found by the PARAFAC model estimation of the spectra. The concentration loadings of the different species during the precipitation, wash, and redissolution process step are depicted in Figures 6C–E, respectively. The VLP species concentration decreases with rising AMS concentration above 1 mol concentration and approaches a limit (see Figure 6C). The VLP concentration loadings of the redissolution step show the inverse behavior above the same threshold (see Figure 6E). The first contaminant cluster shows a similar behavior above 1.5 mol AMS with a higher limit in the precipitation solutions and a lower limit during the redissolution step. Presumably, this contaminant cluster precipitates to the solubility line above the threshold. During redissolution, the precipitate of screened conditions with high AMS concentration is redissolved. The AMS concentration does not strongly affect the concentration loadings of the second contaminant cluster in the precipitation solutions, but the concentration loadings of this component increase slightly to a limit in the redissolution solutions. The second contaminant cluster represents species that are stable at higher AMS concentration. Similar results were achieved by Hillebrandt et al. (2020) for a chimeric VLP construct. The concentration loadings during the wash step show no significant increase in the VLPs and the first contaminant cluster. The second contaminant cluster shows a slight concentration loadings increase and is probably washed out of the precipitate with the rising AMS concentration.

Scanned SDS-PAGE gels of the precipitation and redissolution step are included in the Supplementary Material (see Supplementary Figure S3) analyzing the conditions between 0 mol and 1.27 mol and 2 mol AMS concentration. The findings on the concentration profile of the predicted species match the scanned gel of the reference SDS-PAGE analysis (see Supplementary Figure S3).

**FIGURE 7:**
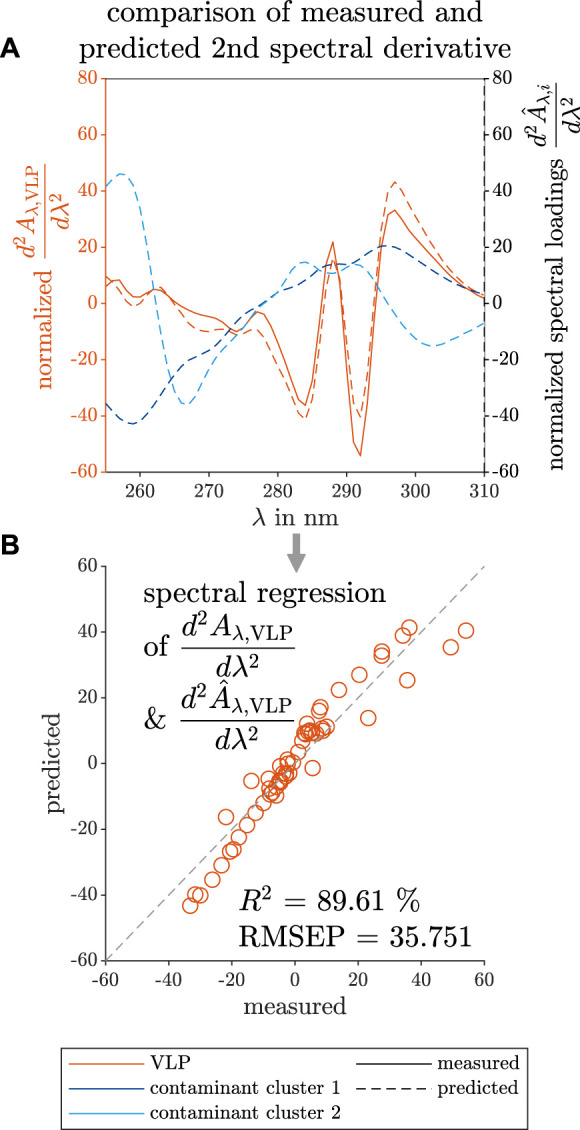
Comparison between predicted and measured data of the spectral loadings of the selective VLP precipitation screening. The measured reference data (left axis) and the predicted loadings (right axis) are illustrated with solid and dashed lines, respectively. The colors orange, dark blue, and light blue indicate the VLPs, and two contaminant clusters. The predictions of the second derivative spectra $\frac{d^{2}{\hat{A}}_{\lambda ,i}}{d\lambda^{2}}$ of the species *i* and the spectral second derivative measurements $\frac{d^{2}A_{\lambda ,VLP}}{d\lambda^{2}}$ of purified VLP solutions are mean-normalized and depicted over the wavelength *λ* in **(A)**. The predicted spectral second derivative loadings of VLPs $\frac{d^{2}{\hat{A}}_{\lambda ,VLP}}{d\lambda^{2}}$ and the reference $\frac{d^{2}A_{\lambda ,VLP}}{d\lambda^{2}}$ are used to plot the predicted over the measured data in **(B)**. The gray dashed line visualizes the ideal fit of the predicted to the measured data. The measured and the predicted spectra are used to calculate the coefficient of determination *R* ^2^ values to quantify the validity of the constructed model.

The similarity between the predicted and measured second derivative of the VLP spectrum is visible in Figure 7A The estimated wavelength position of the peak maxima and minima fits the measured data in the wavelength range below 265 nm and above 275 nm, but the absolute values at the peak maxima and minima do not overlay. Between the mentioned wavelengths, the curve characteristics of the predicted spectral loadings show a flattened curve and differ from the measured data. The absolute values at the peak maxima and minima do not overlay.

This may be the result of the applied preprocessing techniques as smoothing can eliminate or broaden peaks, whereas the spectral derivative calculation is sensitive to subtle differences in spectra.

To visualize the fit of the predicted to the measured data, the mean-normalized predicted VLP loadings and the second derivative data of a measured VLP spectrum are plotted against each other in Figure 7B and used for the calculation of *R* ^2^ and *RMSEP* values. Closer to the center, the predicted data overlay strongly with the measured data. At the boundaries of the spectral loadings, the predicted and the measured data differ more. Still, the spectral loadings showed a high *R* ^2^, but the highest *RMSEP* for the spectral regression among the three investigated case studies.

## 4 Discussion

To prove the overall applicability of PARAFAC models to HT screenings, the three conducted case studies are discussed regarding the choice of the valid PARAFAC model, the process parameters yield and purity, and the differences between the investigated case studies.

### 4.1 PARAFAC model choice

A PARAFAC model can decompose a data set into the signal contribution of each species if the experimental data set has a truly trilinear structure (Bro, 1997; Anzardi et al., 2021). In the case of spectral data sets, this means that an experimental data set can estimate e.g., the spectrum and concentration profile of each species present. Considering the physical logic that the spectra and concentration profiles are positive, the non-negativity constraints can be included in the calculation of chemometric models. This is a common practice to find stable, correct multi-way chemometric models during model calculation (Bro, 1997; Ebrahimi et al., 2008; Murphy et al., 2013; Steiner-Browne et al., 2019; Van Benthem et al., 2020).

Still, valid PARAFAC models can only be constructed if the appropriate number of components (Andersen and Bro, 2003; Bro and Kiers, 2003; Ortiz et al., 2015), preprocessing techniques, and suitable model calculation parameters are used.

In the case of biological, complex solutions containing several different species, the requirement of an appropriate number of PARAFAC components imposes a problem for the model calculation. As not every single UV/Vis-absorbing species can be described by one model component, the different species need to be categorized in clusters. These clusters are formed on the basis of their similar phase behaviors among the species and shall be described by one PARAFAC component accepting inaccuracies in the spectral prediction. This simplification of the variety of species to several clusters introduces an error into the model. However, if the target molecule undergoes a phase transition and contributes strongly to the measured spectral data set, the focus of the PARAFAC models is to find the target molecule in any phase behavior screening study. Further strategies (Smilde et al., 2004) to determine the correct number of PARAFAC components are e.g., half-splitting and comparing the experiments (Bro, 1997), evaluating residuals (Smilde and Doornbos, 1992; Bro, 1997), and the CORCONDIA value (Bro and Kiers, 2003). More information on finding suitable preprocessing (Bro, 1998; Bro and Smilde, 2003) and model calculation parameters (Bro, 1997; Murphy et al., 2013) can be found elsewhere.

In crystallization or precipitation screenings, it can be expected that the protein concentration decreases to the solubility line with increasing precipitant or protein concentration due to the decreased protein solubility, which results in protein crystallization (Asherie, 2004; Baumgartner et al., 2015) or precipitation (Wingfield, 1998; Burgess, 2009; Watanabe et al., 2009). In the case of selective crystallization or precipitation processes, the phase behavior is protein-specific and can be used for protein purification. This theoretical process knowledge can be included in the choice of the PARAFAC model.

The spectral data set for the first case study was recorded for a HT-selective crystallization screening of Lys in a ternary protein system. In total, 96 conditions were screened varying the initial Lys concentration and precipitant concentration. The initial concentrations of the two other proteins (RibA, CytC) were maintained constant in all screened conditions. As the calculation of PARAFAC models with three components did not lead to a robust model, a model with two components was calculated (see Table 1). Evaluating Figure 3A, one component can be identified as the target molecule Lys; the other one as a contaminant cluster resembling mainly CytC. It is assumed that the absorbance contribution of the third species RibA is built into a contaminant cluster (Yang et al., 2015), and that this third species is not described as a single model component. It contributes to a smaller extent to the UV/Vis spectra due to the lower extinction coefficient in the investigated wavelength range (3.8 and 2.8 times lower at 280 nm than for Lys and CytC) and lower concentration (up to 7.5 times lower than the Lys concentration). Furthermore, the protein concentrations of CytC and RibA do not change during the screening, contrary to the target protein Lys (see Wegner et al. (2022) for further explanation). As a consequence, the model cannot distinguish species demonstrating similar phase behavior. This shows that low-absorbing species are difficult to describe with an own model component, and that species with similar phase behavior can be clustered justifying species clustering in screenings with complex solutions.

The selective precipitation study of mAbs leads to a spectral data set, which can be described by a PARAFAC model with three model components (see Table 1). One component represents the target molecule mAb, the other two the AMS concentration and a contaminant cluster. The time profile of the mAb component in Figure 4A may be caused by the changing light refraction when a solution with a high AMS passes the detector (see Subchapter 4.2). Another possible source could be different product-related impurities, e.g., aggregates, fragments, as they would show a mAb resembling spectrum, but different retention times in the analysis system due to diffusion. Below the AMS concentration of 0.5 mol the mAb species is overestimated and the contaminant cluster is underestimated by the PARAFAC model in Figure 4C. In Figure 4E, the two model components show the same effects above 1.4 mol AMS. A possible explanation of these contrasting model discrepancies of the measured to the predicted data is that the predicted mAb UV/Vis spectrum is overestimated below 270 nm leading to inverse effects on the concentration loadings of the mAb and contaminant component. As a result, the spectral loadings of the contaminants may be incorporated in the predicted mAb spectrum and distort the concentration loadings of both species–the target molecule and the contaminant cluster. This effect is more pronounced at higher absorbance values and thus higher protein concentrations.

The protein A chromatography gave further information on the composition of the contaminants during the precipitation, wash, and redissolution step. Figure 5A provides information on the main contaminant cluster during the precipitation and during the redissolution step. This means that the co-precipitated contaminant cluster during redissolution cannot be distinguished from the target molecule.

The PARAFAC model of the selective VLP precipitation HT screening could be calculated with three model components (see Table 1). One component describes the VLP species while the other two describe two contaminant clusters. Assessing the concentration loadings of all three PARAFAC components in Figure 6C, the predicted species show different phase behaviors with increasing precipitant concentration. This enables the use of a selective VLP precipitation step for purification. Regarding the screened redissolution samples in Figure 6E, the predicted concentration loadings of the VLPs and first contaminant cluster increase above the same precipitation threshold in Figure 6C. The second contaminant cluster shows a slight concentration increase at higher precipitant concentration meaning that this cluster was redissolved and thus precipitated at a higher precipitant concentration. This does not comply with the phase behavior during the precipitation step, and it is expected that this discrepancy is caused by model inaccuracies. This assumption is supported by the highest residuals of this model to the measured summed up spectra for the investigated redissolution samples above the stated threshold (data not shown). Overall, the predicted VLP spectral loadings match the measured VLP spectrum (see Figure 7A). Discrepancies are visible in the regression plot (see Figure 7B) only at the higher or lower values of the spectral loadings. Compared to the first and second case studies, the *R* ^2^ value of the third case study for the spectral loadings is lower indicating a greater deviation of the predicted spectra to the measured spectrum. The highest *RMSEP* is partially caused by the different scale and the model mismatch which can be seen in Figure 7B. Additionally, the required preprocessing of the VLP screening data included the second derivative to enhance subtle spectral differences between the screened solutions. The spectral preprocessing may lead to higher discrepancies in Figure 7A and lower accuracy compared to the first and second case studies, but led to a robust model.

In summary, the choice of the correct model component and preprocessing techniques is crucial for the model outcome. These need to be selected with care when the investigated screening solutions involve complex solutions. Theoretical knowledge of selective precipitation and crystallization processes helps finding valid PARAFAC models. Nonetheless, the species in complex solutions demonstrating similar phase behavior can be clustered and described by one model component. In the case of co-precipitation of contaminants with the target molecule, the model may merge the spectra of these species in the predicted spectral loadings.

### 4.2 Screening for optimal yield and purity

The developed models provided information on the solubility line, protein phase behavior, and selectivity of the screened conditions. In the first case study, the solubility line of Lys is visible in the phase diagram in Figure 3B and can be used for further yield calculations. As the concentration of the contaminating species stayed constant in the supernatant, it can be assumed that the produced Lys crystals demonstrate a high purity. The research on mAb crystallization screenings spiked with model protein contaminants showed that a high mAb crystal purity is accompanied by contaminants present in the crystallization supernatant (Zang et al., 2011). In general, this selective crystallization process depends strongly on the impurity and its concentration (Judge et al., 1998; Burke et al., 2001; Liu et al., 2022). Regarding yield, optimal process conditions were achieved in a precipitant range between 0.05 and 0.15 mol AMS.

Assessing the selective mAb precipitation study in Figure 4, a high AMS concentration above 1.8 mol leads to the highest precipitate yield. Under the same precipitant conditions, the concentration loadings of the contaminant species decrease indicating co-precipitation above 1.5 mol AMS, but with a lower yield due to the higher specific solubility concentration. According to the model, the mAb purity of redissolved precipitate is greatly improved when the predicted concentration loadings of the redissolution and the precipitation solutions are compared. Comparing the predicted to measured concentrations, the redissolution solutions show an over- and underestimation of the mAb and contaminant species, respectively. Purity calculations based solely on the predicted concentration loadings would be overestimated. This may be caused by the co-precipitated contaminants (see Figure 5A) as they were not separated during the screening process.

Regarding the selective VLP precipitation process (see Figure 6), the model predicts optimal process parameters when the precipitant concentration lies between 1 mol and 1.5 mol to assure a high purity. The predicted concentration loadings of both contaminant clusters did not indicate co-precipitation and, as a result, are not present in the redissolution samples. To increase the product yield, the concentration above 1.2 mol is desired, as the VLP concentrations in the precipitation and redissolution samples are near the limit. As quantitative reference analytics are missing for the third case study, these results are based purely on model predictions and the qualitative validation with the VLP spectrum and the solution composition with the SDS-PAGE analysis (see Supplementary Figure S3).

### 4.3 Experimental and preprocessing differences between the case studies

The experimental setup and the spectral data preprocessing of each case study required adjustments to the specific protein system. This subchapter focuses on the preprocessing differences between the investigated case study, the experimental screening variations between selective crystallization and selective precipitation studies, and their possible effect on the calculated PARAFAC models.

The time smoothing range for the final models of the crystallization case study was lower than for the precipitation case studies (see Table 1). The four times higher flow rate of the UV/Vis spectral analysis in the first case study is the reason, as the sample passed by the detector in a shorter time (compare Figure 2A, Figure 4A, and Figure 6A) as the time-resolved, spectral information of the sample is comparable between the case studies after preprocessing. Longer time-wise smoothing may lead to the removal of important information for the model calculation. The selected wavelength range for the first case study was broader than for the other two (see Subchapter 2.4.1) since CytC was present in the first case study and has a second absorption maximum at 410 nm.

The third case study required the calculation of the second derivative (see Table 1). Possible reasons could be that the target molecule VLP did not present distinct spectral differences to the contaminants (Mach et al., 1989) or contributed less to the measured spectra compared to target molecules of the first and second case studies. The target protein absorption shares of the initial material was high with 89.24% and 42.82% for the first and second case study, respectively. The VLP absorption share could not be determined as quantitative UV/Vis absorption data as a reference were missing. The large amount of UV/Vis-absorbing contaminants in the VLP lysate may interfere with the identification of the component representing VLPs. The differences in the time profile peak maxima of the target molecules compared to the contaminants support this assumption (see Figure 2A, Figure 4A, and Figure 6A).

For each case study, the buffer system was adapted to the requirements of the target molecule. The buffer substances were not UV/Vis-active in the used concentration and did not affect the model calculation.

On the contrary, the precipitant AMS showed UV/Vis-absorbing behavior in the second case study and had an impact on the constructed models. A possible reason could be that the light refraction occurs when solutions of different density (mobile phase and sample solvent) pass the detector (Raval and Patel, 2020). This strongly depends on the screening AMS concentration and the sample dilution prior to the UV/Vis analysis. In the first case study dealing with the selective crystallization of Lys, the maximal screening AMS concentration was four times lower than in the second and third case studies.

The dilution factors for the first, second, and third case studies varied (see Subchapter 2.2) and were adjusted according to the total absorbance of the initial material at wavelength 280 nm. Taking all these factors into account, the analyzed samples of the second case study (mAb) contained the highest AMS concentration and thus the AMS concentration contributed to a greater extent to the recorded UV/Vis spectra. The constructed model compensated this by describing the precipitant concentration with its own model component (see Figures 4C, D). UV/Vis data recorded of buffer solutions containing different amounts of AMS is shown in Supplementary Figure S1 and support this explanation.

The screening volume, screening size, and the analyzed process step solutions differed. The first case study (Lys crystallization) investigated 96 different conditions in 24 µl batches with eight different Lys starting concentrations and twelve precipitant concentrations. Only the supernatant samples of the crystallization step were analyzed. The spectral data set size was varied in this case study. Screening conditions that did not show concentration changes of the target molecule were excluded for model calculation. It was found that a large screening size with little variety in species composition and concentration ratios does not improve the model robustness but decreases the CORCONDIA value and increases the model error (data not shown). Preferably, the model error is low and the CORCONDIA high indicating an appropriate component number (Bro and Kiers, 2003) and, hence, a valid model. The second and third case studies screened twelve different precipitant concentrations in 500 µl batches for the selective precipitation of mAbs and VLPs. Samples were analyzed during the precipitation, the wash, and the redissolution step leading to a variety of 36 analyzed samples per screening differing in species compositions and concentration ratios. This sample variety improved the model calculation as the CORCONDIA of the final models was higher and the model error lower for the second and the third case studies.

The screening volume did not affect the spectral data set or the model calculation as long as there is enough supernatant for sampling.

When selective crystallization or precipitation processes are characterized with the PARAFAC approach, the models cannot detect if the proteins crystallized or precipitated, as the generated models rely solely on the UV/Vis spectroscopic data set and specific protein concentration reductions. Regarding the experimental differences between the two processes, an additional centrifugation step is required to separate precipitate from the supernatant. Furthermore, the crystallization process requires more time than precipitation processes due to the time-intensive crystal nucleation and crystal growth of macromolecules (Durbin and Feher, 1996; McPherson, 2004).

In summary, these three case studies illustrate how the chemometric multi-way approach of PARAFAC can be applied to different phase behavior screenings with varying process conditions. The differences in spectral data preprocessing could be explained leading to a general preprocessing approach for future crystallization and precipitation screenings. Experimental differences in scale, sample dilution, screening size, and changes of the used chemicals did not interfere with the model calculation as long as the spectra of the target molecule and contaminant species contribute to the UV/Vis spectral measurement and differ in their spectral profiles. A broad variation of the different species concentrations and ratios in the data set was found to be preferred and can be achieved by analyzing different process solutions during selective precipitation or crystallization, washing, and redissolution process steps.

## 5 Conclusion

In this research project, multi-way chemometrics were successfully applied to three high-throughput (HT) screenings for the characterization of selective crystallization and precipitation processes. Supernatant samples were taken after crystallization in the first case study, and after precipitation, washing, and redissolution for the second and third case studies.

Besides model proteins, different modalities, e.g., virus-like particles (VLPs), monoclonal antibodies (mAbs), were investigated. The recorded ultraviolet visible light (UV/Vis) spectra of the samples of each case study were structured as a four-dimensional (4D) data set and preprocessed to eventually calculate one parallel factor analysis (PARAFAC) model per case study. The models of the first and second case studies were compared with quantitative reference data on specific concentrations and spectra of the purified species to test the model validity and to find general preprocessing and model parameters. This knowledge of the calculation parameters was used for the third study when only the spectrum of the purified target molecule could serve as a quantitative reference. The concentration profile was only validated with the qualitative sodium dodecyl sulfate–polyacrylamide gel electrophoresis (SDS-PAGE) analysis.

Without prior calibration, these models coupled with UV/Vis spectroscopy could quickly provide species spectra and concentration estimations for selective crystallization in chemically defined solutions or precipitation screenings in complex solutions.

The calculated PARAFAC components were supposed to represent the various species present in the solution. Still, low-absorbing species or species with similar phase behaviors could not be described with a single model component per species as shown in the first case study. This bears the advantage of clustering species depending on their phase behavior and to better describe multiple impurity species in complex solutions with one model component per cluster. This said, only species which crystallize or precipitate at various precipitant concentrations can be distinguished.

With quantitative insights calculated from the concentration estimations, the generated models could visualize the influence of the precipitant on the different species. Thus, they could be used to evaluate the screened conditions in terms of purity and yield and could potentially find optimal process conditions in all three case studies.

When a suitable model component number was used, reasonable and valid models could be calculated regardless of the modality, screening scale, and other experimental parameters.

This supports the assumption that the approach of coupling PARAFAC and UV/Vis spectroscopy can be transferred to other modalities and purification processes based on phase behavior.

At an exploratory stage of process development, this approach can support process analytical technology (PAT) and it may be especially valuable as deeper process knowledge can be generated without refined analytics and with reduced input of resources. Different impurity clusters and the target molecule can be characterized regarding their differences in spectra and phase behavior. The PAT models estimated yield and purity and can be a basis for detailed process engineering. This process knowledge helps designing selective crystallization and precipitation processes and finding optimal process conditions while complying with the quality by design (QbD) guidelines and the high standard of biopharmaceutical processes.

## Data Availability

The raw data supporting the conclusions of this article will be made available by the authors, without undue reservation.
