# Supplementary material for: Calibration-free PAT: Locating selective crystallization or precipitation sweet spot in screenings with multi-way PARAFAC models
Source: Front Bioeng Biotechnol. 2022 Dec 14;10:1051129. doi: 10.3389/fbioe.2022.1051129 (PMC9797130; doi:10.3389/fbioe.2022.1051129)
Supplement: Supplementary file 1 [file DataSheet1.PDF]

Supplementary Material

1 SUPPLEMENTARY DATA

2 SUPPLEMENTARY TABLES AND FIGURES

2.1 Figures

2.1.1 Case 2 - Selective precipitation of mAbs in a complex solution

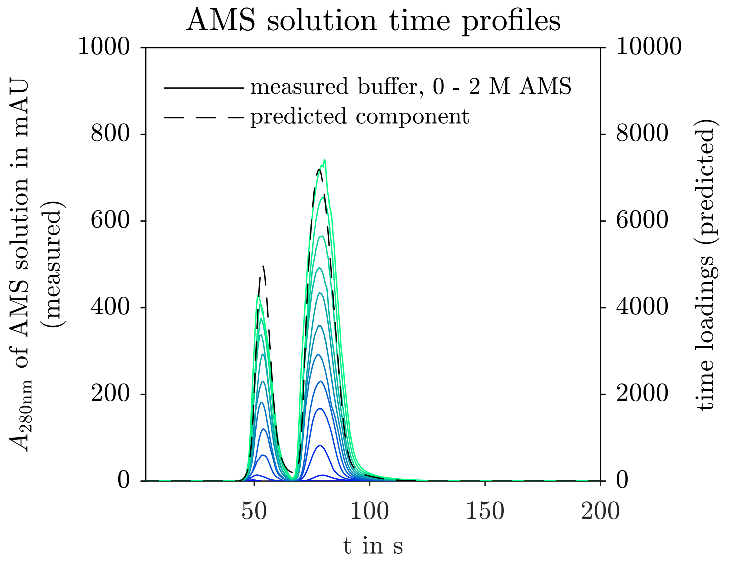

Figure S1. The measured UV/Vis background absorption of the solutions with $0\,\mathrm{M}$ to $2\,\mathrm{M}$ AMS is shown over time with solid lines from blue to green. The predicted time profile of the AMS component for the second case study (mAb) is illustrated with black dashed lines.

Figure S1 illustrates the absorption time profile of AMS solution injections which were diluted as the analyzed samples of the selective mAb precipitation screening. The position of the measured and the predicted time profiles overlay. The peak maximum rises with the AMS concentration of the analyzed sample.

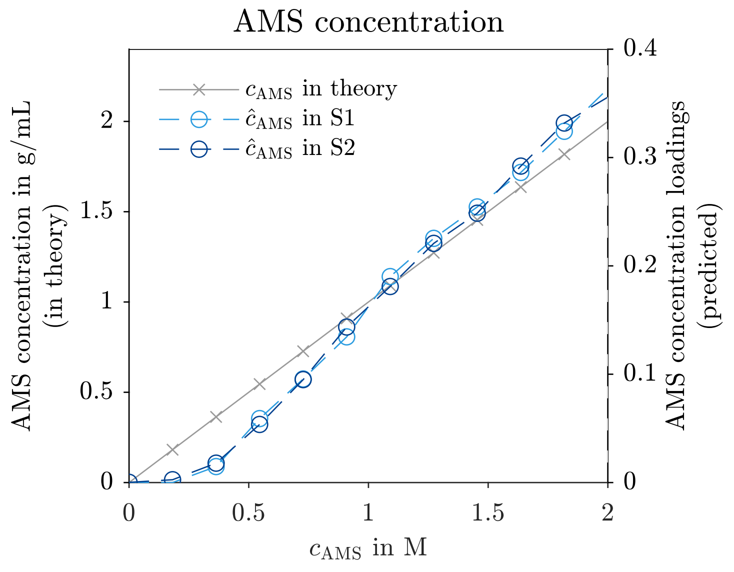

Figure S2. The predicted and the experimental AMS concentration in the precipitation (S1) and wash step supernatant (S2) are shown with blue dashed and gray, solid lines, respectively.

Figure S2 illustrates the AMS concentration during the experiment and from the PARAFAC model. The AMS concentration from the analyzed samples (S1, S2) increases in a linear manner and overlays with the experimental AMS concentration. The authors assume that the discrepancies from the ideal concentration are caused by pipetting or model errors.

2.1.2 Case 3 - Selective precipitation of VLPs in a complex solution

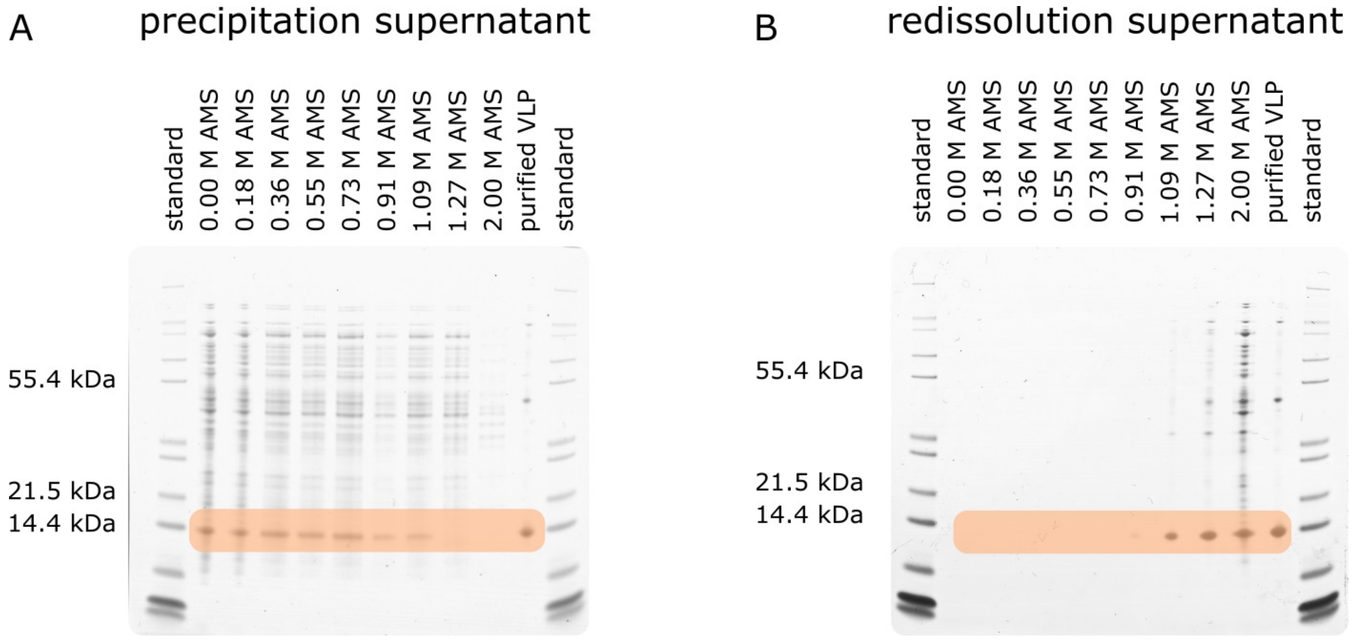

Figure S3. The SDS-PAGE scan of precipitation and redissolution supernatant of selected conditions are depicted in A and B, respectively. A sample of purified VLP was used as a reference to identify the VLP in the gel. The VLP is marked in orange.

Figure S3 shows the scanned SDS-PAGE of precipitation (A) and redissolution supernatant (B) for the selective VLP precipitation screening. The reference data of purified VLPs indicates the position of the target molecule - the VLPs. The solutions with $0\,\mathrm{M}$ to $1.09\,\mathrm{M}$ AMS still contain VLPs, but above $0.73\,\mathrm{M}$ AMS, the band fades. Above $1.27\,\mathrm{M}$ AMS, VLPs are not present in the precipitation supernatant. Species with larger molecular weight remain in the supernatant solution between $0\,\mathrm{M}$ to $1.27\,\mathrm{M}$ AMS which are assumed to be impurities.

The redissolution supernatant solutions indicate that VLPs are present above precipitation conditions above $1.09\,\mathrm{M}$ AMS. Species with larger molecular weight are visible and were redissolved at precipitation conditions above $1.27\,\mathrm{M}$ AMS. The species profile of the precipitation condition with $2\,\mathrm{M}$ AMS indicates a high impurity level as many different species are present. The conditions indicating selective VLP precipitation in the precipitation supernatant agree with the conditions indicating VLP redissolution in the redissolution samples.
